# How Dopamine Enhances an Optimism Bias in Humans

**DOI:** 10.1016/j.cub.2012.05.053

**Published:** 2012-08-21

**Authors:** Tali Sharot, Marc Guitart-Masip, Christoph W. Korn, Rumana Chowdhury, Raymond J. Dolan

**Affiliations:** 1 Department of Cognitive, Perceptual and Brain Sciences, University College London, 26 Bedford Way, London WC1H 0AP, UK; 2 Wellcome Trust Centre for Neuroimaging, Institute of Neurology, University College London, 12 Queen Square, London WC1N 3BG, UK; 3 Institute for Cognitive Neuroscience, University College London, 17 Queen Square, London WC1N 3AR, UK; 4 Department of Education and Psychology, Freie Universität Berlin, Habelschwerdter Allee 45, Raum JK 26/227, 14195 Berlin, Germany; 5 Berlin School of Mind and Brain, Humboldt-Universität zu Berlin, Spandauer Straße 1, 10178 Berlin, Germany

## Abstract

When predicting financial profits [1], relationship outcomes [2], longevity [3], or professional success [4], people habitually underestimate the likelihood of future negative events (for review see [5]). This well-known bias, termed unrealistic optimism [6], is observed across age [7], culture [8], and species [9] and has a significant societal impact on domains ranging from financial markets to health and well being. However, it is unknown how neuromodulatory systems impact on the generation of optimistically biased beliefs. This question assumes great importance in light of evidence that common neuropsychiatric disorders, such as depression, are characterized by pessimism [10, 11]. Here, we show that administration of a drug that enhances dopaminergic function (dihydroxy-L-phenylalanine; L-DOPA) increases an optimism bias. This effect is due to L-DOPA impairing the ability to update belief in response to undesirable information about the future. These findings provide the first evidence that the neuromodulator dopamine impacts on belief formation by reducing negative expectations regarding the future.

## Highlights

► L-DOPA impairs ability to update beliefs in response to undesirable information ► Enhancing dopamine function reduces negative expectations regarding the future

## Results

Humans are optimistically biased when making predictions about the future, habitually underestimating the likelihood of negative events [1–8]. This bias is related to a striking asymmetry whereby people update their beliefs more in response to information that is better than expected compared to information that is worse than expected [12, 13]. Selective updating is mediated by regions of the frontal cortex that track errors in estimation when these call for positive update but show a relative failure to code for errors that might induce a negative update [12].

An unresolved question is whether neuromodulators associated with generating expectations of future outcomes influence this process. A prominent candidate is the monoamine dopamine, a neuromodulator suggested to provide a teaching signal that indexes when predictions fail to align with outcomes [14, 15]. In Parkinson's disease, drugs enhancing dopaminergic function (e.g., dihydroxy-L-phenylalanine; L-DOPA) influence learning of positive and negative outcomes in an asymmetric manner, enhancing the former and impairing the latter [16]. Dopamine effects on learning have been extensively studied in the context of model-free reinforcement learning [14–16]. However, it also impacts on domains as diverse as working memory, episodic memory, and reversal learning [17, 18]. Given these set of findings [12, 16], we hypothesize that enhancing dopamine function will influence how healthy individuals incorporate information about probabilities of future life events in an asymmetric manner, increasing an optimism bias.

**Figure 1:**
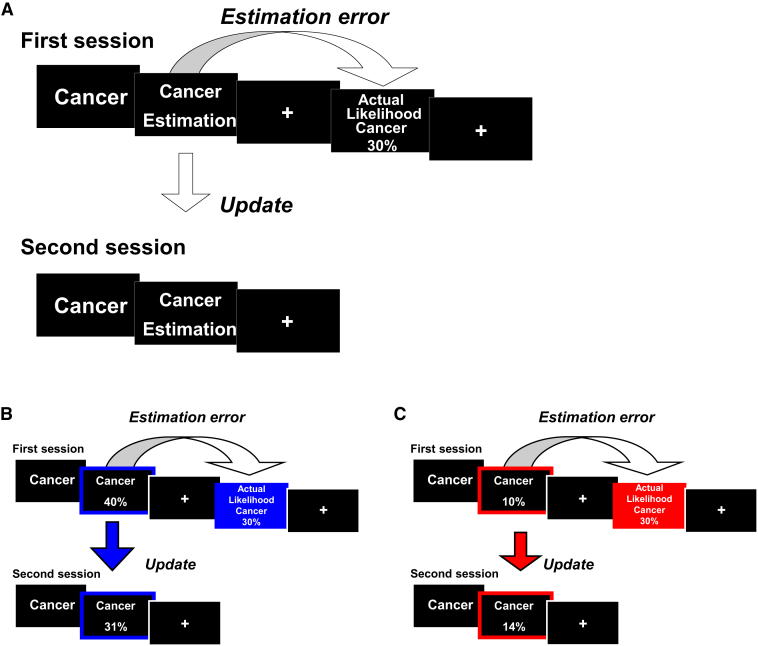
Task Design (A) On each trial, participants were presented with a short description of one of 40 adverse life events and asked to estimate how likely this event was to occur to them. They were then presented with the average probability of that event occurring to a person like themselves, living in the same sociocultural environment. For each event, an estimation error term was calculated as the difference between the participant's estimation and the information provided. The second session was the same as the first session. For each event, an update term was calculated as the absolute difference between the participant's first and second estimations. Participants completed both sessions twice on two separate days, with different stimuli, once under placebo and once after the administration of the drug. (B and C) Examples of trials for which the participant's estimate was higher (B) or lower (C) than the average probability. Here, for illustration purposes, the blue and red frames denote the participant's response (either an overestimation or underestimation, respectively). For illustration purposes, the blue and red filled boxes denote information that calls for an adjustment in an optimistic (desirable) (B) or pessimistic (undesirable) (C) direction.

To test whether an optimism bias is modulated by dopamine, participants completed a belief update task [12] on two separate days, one week apart (Figure 1), in a double-blind placebo-controlled pharmacological intervention study. On one of the days, participants received placebo and on the other they received L-DOPA (150 mg), in a counterbalanced order (n = 21). The task was identical on both days except for the fact that different stimuli were used on each day (lists were counterbalanced). At each session, participants provided estimates of their likelihood of experiencing 40 different types of adverse life events (e.g., Alzheimer's disease, robbery; see the List of Stimuli in the Supplemental Experimental Procedures available online) adapted from a previous study [12]. After each trial, they were presented with an actuarial average probability of that event occurring to a person from the same sociocultural environment. We then assessed whether participants used this information to update their predictions by subsequently asking them to again estimate their likelihoods for the same 40 events in a second session, taking place ∼15 min after the first session. They also completed a memory test for the information presented and rated all stimuli on different subjective scales (for a full description, see Supplemental Experimental Procedures).

To test whether effects might be observed when manipulating another neuromodulator implicated in learning about reward and punishment, we administered the serotonergic reuptake inhibitor citalopram (24 mg in oral drops, equivalent to 30 mg in tablets) to a second group of participants (n = 19). Serotonin neurotransmission is suggested to be involved in aversive processing and inhibition ([19, 20], but see [21]). However, the nature of its role in learning is less established than is the case for dopamine.

### Optimism Bias Grows with Increased Dopamine Function

We found that enhancing participants' dopamine function increased their prediction bias in an optimistic direction. Specifically, for each participant on each trial, we subtracted the participant's estimation of how likely they were to encounter the negative event from the average probability of encountering that event (i.e., estimation error = estimation − probability presented). If the average estimation error was negative, then this indicated that participants tended to underestimate their likelihood of encountering aversive events relative to the average probability in the population (optimistically biased predictions). A positive number indicated a bias in a pessimistic direction, and a score of zero indicated that the extent of overestimation is equal to that of underestimation. Note that if the average estimation error is small in magnitude, this does not imply that the subject is accurate in their estimation; rather, it reflects that they are not biased in any particular direction.

**Figure 2:**
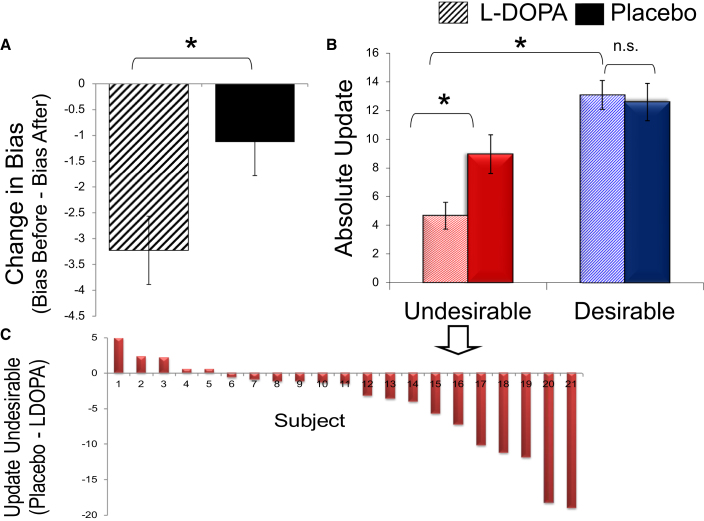
L-DOPA Enhances the Optimism Bias by Impairing Update for Undesirable Information (A) Change in bias is equal to the bias after participants were presented with the average probability minus the bias before. Bias is equal to the signed estimation errors (= participant's estimate of the likelihoods of encountering future adverse events − average probability presented). Note that the extent of the bias does not signify accuracy. A score of zero indicates that the extent of overestimation is equal to the extent of underestimation (i.e., errors are not biased in any direction). A negative score indicates bias is optimistic; a positive score indicates bias is pessimistic. Administration of L-DOPA enhanced the change in bias toward an optimistic direction (after controlling for differential memory and differential scores on all rating scales). See also Table S1. (B) Absolute update (participant's second estimate − first estimate) for trials where participants received desirable information that presented an opportunity to adopt a more optimistic outlook, and for trials where they received undesirable information calling for a more pessimistic estimate. Update in response to undesirable information was reduced in the L-DOPA condition relative to placebo. (C) The decrease in updating from negative information in the L-DOPA condition relative to placebo is shown for all subjects. Subjects are ordered according to the magnitude of the effect (ascending). Error bars represent SEM corrected for within-subject design [22]. ^∗^p < 0.05.

For each subject, the change in average estimation errors (i.e., the change in bias = bias after presentation of information − bias before the presentation of information) was calculated and compared using a one-way analysis of covariance (ANCOVA) with condition (drug/placebo) as factor and differential scores on all subjective scales and memory controlled for by entering them as covariates (see Supplemental Experimental Procedures for details). The change in bias in the L-DOPA condition was greater than in the placebo condition [F(1,14) = 9.37, p < 0.01] (Figure 2A). As shown in Figure 2A, the change in bias in the L-DOPA condition was due to an effect in the optimistic direction and significantly different from zero [t(20) = 2.99, p < 0.01].

The change in bias in the citalopram condition was not different from in the placebo condition [F(1,12) = 0.01, p > 0.9]. We note that no assays of plasma drug levels or changes in endocrine measures were documented, which might have shown that the drug levels were not adequate to produce significant changes.

### L-DOPA Impairs Updating in Response to Undesirable Information Regarding the Future

We next investigated whether L-DOPA increased the optimism bias by enhancing learning from desirable information regarding the future, decreasing learning from undesirable information, or both. To this end, as implemented previously [12], we divided trials into those for which the average probability of experiencing a negative life event was better than the participants' own probability estimate (i.e., trials for which a subject received desirable information) relative to a situation where the average probability was worse (i.e., subjects received undesirable information). The average degree of absolute belief update was then computed for each participant and condition (update = |participant's second estimate − participant's first estimate|). We found that L-DOPA selectively impaired belief update in response to undesirable information about the future (Figure 2B). Specifically, a 2 (condition: L-DOPA/placebo) by 2 (valence: desirable/undesirable) ANCOVA on update scores revealed a significant interaction [F(1,14) = 7.4, p < 0.02]. The interaction was characterized by a reduction in update magnitude in response to undesirable information under L-DOPA relative to placebo [t(20) = 3.03, p < 0.01, observed in 76% of the participants; Figure 2C], but there was no significant difference in response to desirable information under L-DOPA relative to placebo [t(20) = 0.34, p > 0.7]. Thus, selective updating, whereby participants update their beliefs when receiving desirable information relative to undesirable information, was enhanced by L-DOPA.

**Figure 3:**
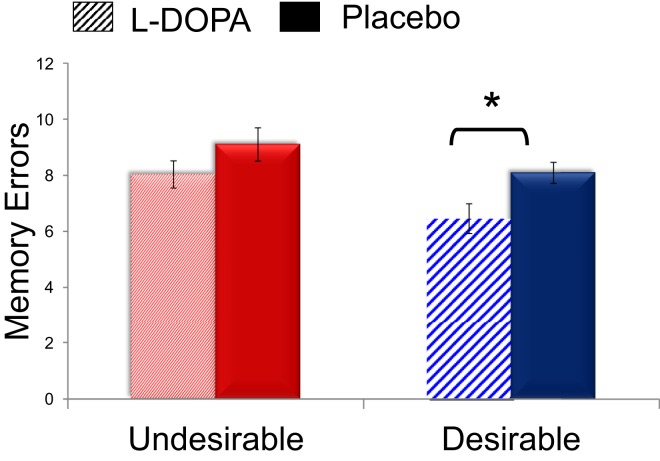
Memory for Information Provided Memory performance for the information provided was calculated as the absolute difference between the statistical number presented for each event and the participants' recollection of that number. These memory errors are presented for trials where participants received desirable information that presented an opportunity to adopt a more optimistic outlook, and for trials where they received undesirable information calling for a more pessimistic estimate, in the L-DOPA and placebo conditions. Error bars represent SEM corrected for within-subject design [22]. ^∗^p < 0.05. See also Table S1.

Our findings were not explained by valence-dependent effects of L-DOPA on memory (see Supplemental Experimental Procedures for a full description of how memory scores were calculated), emotional arousal, extent of negative valence, sense of familiarity, or sense of past experience with the adverse life events, because all these variables were added as covariates in all ANCOVAs (see Table S1 for scores on all variables and Supplemental Experimental Procedures for a description of all scales). Note that L-DOPA tended to increase memory for the information provided [F(1,20) = 3.67, p = 0.07], an effect that reached significance for desirable information only [desirable: t(20) = 2.12, p < 0.05; undesirable: t(20) = 1.2, p > 0.1], although there was no interaction [F(1,20) = 0.46, p > 0.5; Figure 3]. Furthermore, L-DOPA did not cause side effects, nor did it affect mood or reaction times (see Tables S1 and S2).

We observed no effect of citalopram on updating [main effect of condition: F(1,12) = 1.7, p > 0.6; interaction between condition and valence: F(1,12) = 0.8, p > 0.8]. Although citalopram did not impact on updating, this does not imply that citalopram, or serotonin function, does not influence optimism. An optimism bias can in principle be generated via a number of different mechanisms [5, 12, 23], and it is possible that serotonin could impact on expectations by altering other processes, such as mental simulation of future events [23] or increased memory for positive emotions [24].

## Discussion

We show that a tendency to incorporate undesirable information into one's forecasts of the future is impaired when dopamine levels are enhanced. This leads to an underestimation of the likelihood of negative events, a fundamental characteristic of unrealistic optimism. Previously, we reported that increasing dopamine function while people imagined positive future events enhanced expectations of pleasure to be derived from those events [25]. Whereas dopamine in our previous study altered hedonic expectancies, in the current study we show that enhancing dopamine influences positive expectations by attenuating the impact of unexpected negative information. In this regard, our findings converge with observations from patients with Parkinson's disease where enhanced dopaminergic levels lead to impaired learning from unwanted outcomes [16]. Although the latter study differed significantly from ours, not only in terms of the target population (Parkinsonian patients rather than healthy individuals) but also in relation to a requirement to learn from actual outcomes (rather than information of possible future outcomes) in the context of a reinforcement learning task, we suggest that the underlying mechanisms may be shared. Specifically, L-DOPA administration may interfere with dopamine dips, the putative mechanism signaling a worse-than-expected outcome [26, 27] suggested to support learning from negative prediction errors [16, 28]. We speculate that dopamine modulation of frontal cortex function may explain the current results, given that dopamine plays a modulatory role in cognition through its extensive diffuse projections from midbrain dopamine nuclei to the basal ganglia and frontal cortical areas [29–31].

Note that we do not conclude that the effect of L-DOPA is specific to learning about possible future outcomes (differential effects of dopamine on the impact of positive and negative outcomes may be general). Instead, the relevance of our findings is in providing an explanation of how dopamine enhances unrealistic optimism. Interestingly, our results also hint at a possible mechanism by which antidepressant medication that targets dopamine function might reduce depressive symptoms and cognitions [32]. A core symptom profile in major depression disorder is pessimistic beliefs [10]. Individuals with mild depression exhibit less biased expectations than healthy individuals, whereas those with severe depression express a pessimistic bias [11]. By reducing the probability of updating expectations in response to negative information, medication that enhances dopamine levels might lead to a diminution of a pessimistic outlook in depressed patients.

Understanding how predictions of future life events are altered is critical for understanding human action and decision making, which is largely driven by predictions of likely negative and positive outcomes [33–35]. It is also key to understanding how expectations go awry in neuropsychiatric disorders where dopamine function is an implicated mechanism, including depression [32], addiction [36], and attention deficit hyperactivity disorder [37]. Our data provide novel evidence that pharmacological manipulation of the neurotransmitter dopamine alters the efficiency of human belief updating, reducing negative expectations in a manner that leads to a boosting of unrealistic optimism.

## Experimental Procedures

### Participants

Forty participants were recruited via the University College London psychology subject pool. Participants were randomly assigned to either the L-DOPA condition (n = 21, mean age 23.9 years, 11 females, 10 males) or the citalopram condition (n = 19, mean age 22.5 years, 10 females, 9 males). The study was double blind. All subjects gave informed consent and were paid for their participation. They completed a screening form for significant medical conditions, signed a form declaring that they were not receiving other medications or using illicit drugs, and were paid for their participation.

### Stimuli

Eighty short descriptions of negative life events (e.g., car theft, Parkinson's disease; see the List of Stimuli in the Supplemental Experimental Procedures) were presented. Stimuli were split into two lists of 40 events each. One list was used on day 1 and the other on day 2, randomly assigned.

For each adverse event, the average probability of that event occurring at least once to a person living in the same sociocultural environment as the participants was determined based on online resources. Very rare or very common events were not included; all event probabilities lay between 10% and 70%. To ensure that the range of possible overestimation was equal to the range of possible underestimation, participants were told that the range of probabilities was between 3% and 77%.

### Procedure

Participants completed the task on two days one week apart. On one day they received placebo, and on the other day either L-DOPA (150 mg) together with benserazide (37.5 mg, which promotes higher levels of dopamine in the brain while minimizing side effects from peripheral dopamine, such as nausea and vomiting) 2 hr before task completion, or citalopram (24 mg in drops, which is equivalent to 30 mg in tablet) 4 hr before task completion; order was randomly assigned and counterbalanced. L-DOPA's half-life is ∼1.5 hr (peaks at 1 hr) and citalopram's is ∼35 hr (peaks at 4 hr).

After completing the main task, subjects completed the memory test and additional ratings (see Supplemental Experimental Procedures) and a subjective state questionnaire [38] (see Table S1 for scores). Before completing the task, subjects participated in a separate fMRI study using a go/no-go task [39]. There is no a priori theoretical link between the two studies, and the reason for using the same subjects in both tasks was purely efficiency.

### Task

On each trial, a stimulus was presented on screen for 2 s. During that time, participants were instructed to think of that event happening to them in the future. When the words “Estimation of happening?” appeared on screen they were to estimate how likely the event was to happen to them in the future. Participants had up to 6 s to respond using the keyboard. If the participant failed to respond, that trial was excluded from all consequent analyses. A fixation cross then appeared for 1–3 s (jittered). Next, the event description appeared again for 2 s together with the average probability of that event occurring. Finally, a fixation cross appeared for 1–3 s (jittered). Immediately after completing 40 trials, participants estimated all events again. The procedure was the same as above, except that the average probability of the event occurring was not presented again.

## Supplemental Information

Document S1. Supplemental Experimental Procedures and Tables S1 and S2
