# Supplementary material for: How Dopamine Enhances an Optimism Bias in Humans
Source: Curr Biol. 2012 Aug 21;22(16):1477–81. doi: 10.1016/j.cub.2012.05.053 (PMC3424419; doi:10.1016/j.cub.2012.05.053)
Supplement: Document S1. Supplemental Experimental Procedures and Tables S1 and S2 [file mmc1.pdf]

Current Biology, Volume *22*

Supplemental Information

How Dopamine Enhances an Optimism Bias in Humans

Tali Sharot, Marc Guitart-Masip, Christoph W. Korn, Rumana Chowdhury, and Raymond J. Dolan

Supplemental Experimental Procedures

Main Behavioural Analysis

Bias - For each event in each session a signed *estimation error* term was calculated as the difference between the participant's *estimate* and the corresponding statistical *probability presented*.

(1) *Bias = estimation error = first estimate –probability presented*

For each participant the average estimation error was calculated for session 1 (before information was presented) and for session 2 (after information was presented) for the placebo and drug condition separately. A positive number indicates that the subject has a pessimism bias (i.e. the errors are biased towards overestimating the probability of encountering a negative event). A negative number indicates that the subject has an optimism bias (i.e. the errors are biased towards under-estimating the probability of encountering a negative event). Zero indicates that the subject does not have a bias in any particular direction (note, that this does not indicate accuracy).

We then calculated the change in bias before and after information was presented:

(2) *Change in bias = Bias after information was presented – Bias before information was presented*

Update - The amount of *update* was calculated as the absolute difference between the first and second estimates.

(3) *update* = │first *estimate* – second *estimate*│

For each participant, average absolute update scores in each condition (drug /placebo) were calculated for trials where the participant received desirable and undesirable information.

Note that in our previous study we showed that differential updating following desirable and undesirable information was not explained by differential processing of high and low numbers [12]. In that study participants were asked to estimate their likelihood of encountering the adverse event on half the trials, and to estimate their likelihood of not encountering the adverse event on the rest of the trials. The wording of the task did not affect any of the results [12].

Memory Test and Analysis

After the main task participants indicated the actual probability (previously presented) of each event occurring to an average person in the developed world. Memory errors were calculated as the absolute difference between the actual probability previously presented and the participant's recollection of that statistic.

Memory error = │*Actual probability presented – Recollection of probability presented*│.

For each participant, average memory scores in each condition (drug, placebo) were calculated for trials where the participant received desirable and undesirable information. Differential memory scores (desirable minus undesirable) were added as covariates in all ANCOVAs.

Additional Rating Scales

Participants were presented with the same trials again on a computer screen and were asked to rate events on five scales: Vividness (How vividly could you imagine this event? From 1 = not vivid to 6 = very vivid); Familiarity (Regardless if this event has happened to you before, how familiar do you feel it is to you from TV, friends, movies and so on? From 1 = not at all familiar to 6 very familiar); Prior experience (Has this event happened to you before? From 1 = never to 6 = very often); Arousal (When you imagine this event happening to you how emotionally arousing is the image in your mind? From 1 = not arousing at all to 6 = very arousing); and Negativity (How negative would this event be for you? From 1 = not negative at all to 6 = very negative). For each participant, average scores for each scale in each condition (drug/placebo) were calculated for trials where the participant received desirable and undesirable information. These scores and the relevant statistics are presented in Table S2.

Differential scores (desirable – undesirable) on all 5 scales were added as covariates in all ANCOVAs.

List of Stimuli

fraud when buying something on the internet
theft from vehicle
card fraud
sport related accident
household accident
mouse/rat in house
knee osteoarthritis (causing knee pain and swelling)
being cheated by husband/wife
more than £30,000 debts
miss a flight
hernia (rupture of internal tissue wall)
death before 80
witness a traumatising accident
domestic burglary
bone fracture
depression
heart failure
obesity
irritable bowel syndrome (disorder of the gut)
chronic high blood pressure
diabetes (type 2)
victim of violence by stranger
disease of spinal cord
serious hearing problems
infertility
car stolen
dementia
drug abuse
gallbladder stones
being convicted of crime
house vandalised
restless legs syndrome
gluten intolerance
appendicitis
age related blindness
genital warts
chronic ringing sound in ear (tinnitus)
death before 60
alcoholism
Parkinson's disease
back pain
computer crash with loss of important data
being fired
eye cataract (clouding of the lens of the eye)
skin burn
hospital stay longer than three weeks
bicycle theft
divorce
victim of bullying at work (nonphysical)
arteries hardening (narrowing of blood vessels)
theft from person
having fleas/lice
sexual dysfunction
hepatitis A or B
victim of violence with need to go to A&E
severe teeth problems when old
cancer (of digestive system/lung/prostate/breast/skin)
abnormal heart rhythm
victim of violence by acquaintance
herpes
migraine
having a stroke
victim of violence at home
severe insomnia
osteoporosis (reduced bone density)
death before 70
severe injury due to accident (traffic or house)
autoimmune disease
artificial joint
victim of mugging
asthma
blood clot in vein
ulcer
kidney stones
Alzheimer's disease
anxiety disorder
limb amputation
epilepsy
liver disease
death by infection

Events Used During the Training Sessions

dying before 90
glaucoma
post traumatic stress disorder

Table S1. Subjective Scales, Memory, RT

	L-DOPA - Placebo		Citalopram - Placebo	
**Subjective Scales Questionnaire**	**Undesirable**	**Desirable**	**Undesirable**	**Desirable**
Vividness (1) low - (6) high	-0.14	0.11	-0.41	-0.27
Familiarity (1) low - (6) high *	0.01	0.28	-0.05	0.01
Past Experience (1) low - (6) high*	-0.12	0.11	-0.02	0.01
Arousal (1) low - (6) high*	-0.02	0.34	-0.11	-0.18
Negative (1) low - (6) high*	-0.01	0.30	0.21	0.11
Memory Errors	-1.07	-1.64^	-1.41	-0.08
Reaction Time First Estimate (ms)	146.99	49.27	11.54	24.10
Reaction Time Second Estimate (ms)	8.03	-40.59	-50.78	-3.20

Scores represent difference in subjective ratings, memory errors and reaction times between drug condition and placebo condition.
No significant differences detected between Citalopram and Placebo.
Difference between L-DOPA and placebo are represented as follows:
*****Interaction between condition (drug/placebo) and valence (desirable/undesirable) in L-DOPA condition, $P < 0.05$.
^Difference between drug and placebo in either the desirable or undesirable condition, $P < 0.05$.

Table S2. Subjective State Questionnaire

	Change in Ratings	
**Subjective State Questionnaire**	**L-DOPA - Placebo**	**Citalopram- Placebo**
Alert (1) - Drowsy (6)	0.60	1.15
Calm(1) - Excited (6)	0.53	-0.15
Strong(1) - Feeble (6)	0.49	0.11
Muzzy (1) -Clear Headed (6)	-1.10	-0.50
Well Coordinated (1) - Clumsy (6)	-0.02	-0.11
Lethargic (1) - Energetic (6)	-0.12	-0.42
Contented (1) - Discontented (6)	0.30	0.34
Troubled (1) - Tranquil (6)	-0.17	-0.14
Mentally slow (1) - Quick witted (6)	1.32	-0.15
Tense (1) - Relaxed (6)	-0.55	-0.65
Attentive (1) - Dreamy (6)	-0.78	-0.45
Incompetent (1) - Proficient (6)	0.16	-0.07
Happy (1) - Sad (6)	0.05	-0.23
Antagonistic (1) - Friendly (6)	0.09	0.61
Interested (1) - Bored (6)	-0.45	0.01
Withdrawn (1) - Sociable (6)	0.09	0.20

Scores represent difference in ratings between drug condition and placebo condition. Neither L-DOPA nor Citalopram had significant effects on any measures of subjective state. 3 Subjects in the L-DOPA condition and 4 in the Citalopram condition did not complete the questionnaire in either day 1 or day 2.
